# Association of TyG index and TG/HDL-C ratio with arterial stiffness progression in a non-normotensive population

**DOI:** 10.1186/s12933-021-01330-6

**Published:** 2021-07-06

**Authors:** Zhiyuan Wu, Di Zhou, Yue Liu, Zhiwei Li, Jinqi Wang, Ze Han, Xinlei Miao, Xiangtong Liu, Xia Li, Wei Wang, Xiuhua Guo, Lixin Tao

**Affiliations:** 1 Beijing Municipal Key Laboratory of Clinical Epidemiology, Department of Epidemiology and Health Statistics, School of Public Health, Capital Medical University, Beijing, China; 2 Department of Mathematics and Statistics, La Trobe University, Melbourne, Australia; 3 School of Medical and Health Sciences, Edith Cowan University, Perth, Australia

**Keywords:** Insulin resistance, Arterial stiffness, Hypertension, Triglyceride glucose index, TG/HDL-C ratio, Cohort study

## Abstract

**Background:**

Cross-sectional studies have reported that insulin resistance (IR) is associated with arterial stiffness. However, the relationship between IR and arterial stiffness progression remains unclear. This study aims to evaluate the association of triglyceride glucose (TyG) index and triglyceride to high-density lipoprotein cholesterol (TG/HDL-C) ratio with arterial stiffness progression in a non-normotensive population.

**Methods:**

A total of 1895 prehypertensive (systolic pressure 120–139 mmHg or diastolic pressure 80–90 mmHg) or hypertensive (systolic pressure ≥ 140 mmHg or diastolic pressure ≥ 90 mmHg or using antihypertensive medication) participants were enrolled in 2013 and 2014, and followed until December 31, 2019. Arterial stiffness progression was measured by brachial-ankle pulse wave velocity (baPWV) change (absolute difference between baseline and last follow-up), baPWV change rate (change divided by following years), and baPWV slope (regression slope between examination year and baPWV).

**Results:**

During a median follow-up of 4.71 years, we observed an increasing trend of baPWV in the population. There were linear and positive associations of the TyG index and TG/HDL-C ratio with the three baPWV parameters. The difference (95% CI) in baPWV change (cm/s) comparing participants in the highest quartile versus the lowest of TyG index and TG/HDL-C ratio were 129.5 (58.7–200.0) and 133.4 (52.0–214.9), respectively. Similarly, the evaluated baPWV change rates (cm/s/year) were 37.6 (15.3–60.0) and 43.5 (17.8–69.2), while the slopes of baPWV were 30.6 (9.3–51.8) and 33.5 (9.0–58.0). The observed association was stronger in the hypertensive population.

**Conclusion:**

Our study indicates that the TyG index and TG/HDL-C ratio are significantly associated with arterial stiffness progression in hypertensive population, not in prehypertensive population.

**Supplementary Information:**

The online version contains supplementary material available at 10.1186/s12933-021-01330-6.

## Background

Hypertension has caused a heavy economic burden worldwide, becoming a challenging public health issue. In 2010, 31.1% of adults around the world were reported to have hypertension [1]. Among Chinese adults aged 35–75 years, nearly half are diagnosed with hypertension, the incidence is still steadily increasing, and the onset age is becoming younger [2]. Among hypertensive patients, arterial stiffness is a common vascular complication and is also an independent risk factor and predictor of other cardiovascular and cerebrovascular diseases [3, 4], such as coronary heart disease (CHD) and stroke. Therefore, it is of great importance to focus on the progression of arterial stiffness in the hypertensive population and identify the early related factors of arterial stiffness [5–7].

Disorders of glucose and lipid metabolism are a common pathophysiological feature accompanying patients with hypertension, while insulin resistance (IR) extensively participates in this biological process [8]. The hyperinsulinaemic-euglycaemic clamp is the gold standard for evaluating the status of IR [9]. However, this assessment process is expensive and complex and is not ideal for routine clinical monitoring. Recently, some novel and simple indicators have been reported to be reliable surrogate indexes of IR, such as the triglyceride-glucose (TyG) index and the triglyceride to high-density lipoprotein cholesterol (TG/HDL-C) ratio. Compared with the hyperinsulinaemic-euglycaemic clamp, the TyG index has a high sensitivity of 96.5% and a specificity of 85.0% for the diagnosis of IR [10], which has also been reported to be associated with diabetes in Chinese population [11]. Many studies have found that these surrogate indexes are independent risk factors for some cerebrocardiovascular diseases [12–14]. Moreover, IR-related indexes are associated with arterial stiffness [15–19]. However, these studies are all based on general populations. Recently, Li et al. [20] found that the TyG index is positively associated with brachial-ankle pulse wave velocity (baPWV) in hypertensive patients. However, this study is a cross-sectional study without follow-up. Moreover, the association between the TG/HDL-C ratio and arterial stiffness remains unreported.

Therefore, we aimed to comprehensively investigate the association between the TyG index and TG/HDL-C ratio with arterial stiffness progression in prehypertensive and hypertensive populations based on a prospective cohort study.

## Methods

### Study design and participants

**Fig. 1:**
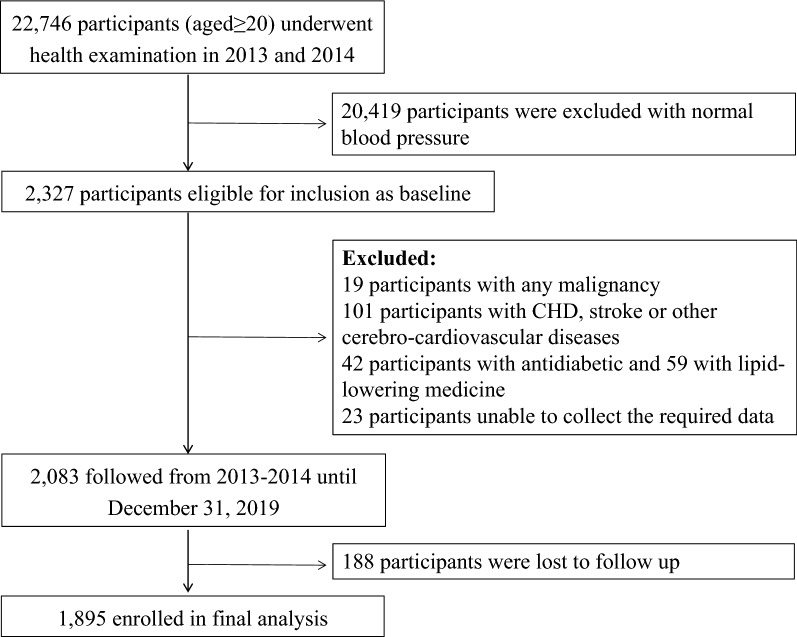
Flow chart of the study population

The Beijing health management cohort (BHMC) is an open cohort study established in 2008 in Beijing, China, with new individuals recruited annually. The BHMC study was conducted based on health examination populations from the Beijing Xiaotangshan Examination Center and Beijing Physical Examination Center. BHMC was designed to investigate the risk factors and biomarkers for metabolism-related diseases, and the recruited individuals were asked to take an annual health examination, including physical examination (anthropometry variables, blood pressures), face-to-face questionnaire survey (demographic variables, lifestyles, diseases history, medication history) and biochemical examination. Details of the study design have been described previously [21]. The arterial stiffness-related variables, such as baPWV and ankle brachial index (ABI), were available from 2013. Therefore, individuals taking health examinations in 2013 and 2014 were recruited in this current study as a baseline. Of 22,746 participants who underwent health examination at baseline, 2327 were confirmed to have prehypertension or hypertension. To minimize the possible influence of medication on glucose and lipid levels, 42 participants using antidiabetic agents and 52 participants using lipid-lowering agents were excluded. In addition, we excluded 101 participants with CHD, stroke or other cerebrocardiovascular diseases. Nineteen participants with any malignancy were excluded. Twenty-three participants from whom we were unable to collect the required data at baseline and 188 participants lost to follow-up, defined as without health examination records until 2019, were further excluded from the analyses. Finally, this study was restricted to a subset of 1895 participants with complete data, which were used in the final analyses, as shown in Fig. 1. Most of the participants (98%) enrolled in this current study came from a work unit in Beijing, and there were a total of 407 units included.

This study was in accordance with the principles of the Declaration of Helsinki and approved by the Ethics Committee of Capital Medical University (grant number: 2020SY031). All participants provided written informed consent before taking part in this study.

### Data collection and definitions

The demographic characteristics, lifestyle, and medication-related information were collected via a standard questionnaire by our trained staff, including age, sex, occupation, smoking status, drinking status, regular physical activity, diagnosis history of diseases and medication information. Occupation in this current study was classified into ‘manager’, ‘technician or professional’, ‘worker’ and ‘retired’. Smoking status was defined as ‘current smoker’, ‘former smoker’ and ‘never smoked’. Drinking status was defined as ‘current drinking’ and ‘no current drinking’. Physical activity was defined as having moderate or intense exercise ‘ ≥ 80 min per weak’ and ‘ < 80 min per week or none’. The physical and biochemical examination data were acquired from the electronic medical record system. Body mass index (BMI) was calculated as weight (in kilograms)/height^2 (in metres squared). Systolic blood pressure (SBP) and diastolic blood pressure (DBP) were presented as the average of two measurements on the right arm using a sphygmomanometer after resting for at least 10 min. Based on the JNC-7 criteria [22], hypertension status was defined as SBP ≥ 140 mmHg or DBP ≥ 90 mmHg or use of any antihypertensive medication. Prehypertension status was defined as SBP of 120–139 mmHg or DBP of 80–89 mmHg. The mean arterial pressure (MAP) was calculated as 1/3*SBP + 2/3*DBP. A higher vascular stiffness increases pulse pressure (PP), while PP could increase SBP and decrease DBP to a small extent. Therefore, the MAP level was adjusted in the following analyses.

Blood samples were stored and measured in the central laboratory of Beijing Xiaotangshan Examination Center or Beijing Physical Examination Center using the Olympus Automatic Biochemical Analyser (Hitachi 747; Tokyo, Japan). Serum total cholesterol (TC), triglyceride (TG), high-density lipoprotein cholesterol (HDL-C), and low-density lipoprotein cholesterol (LDL-C) were measured with the enzymatic colour metric method. The estimated glomerular filtration rate (eGFR) was calculated using the Chronic Kidney Disease Epidemiology Collaboration (CKD-EPI 2009) serum creatinine equation [23]. Fasting blood glucose (FBG) was defined as the glucose concentrations before breakfast after overnight fasting (no food, except drinking water, for at least 8–10 h), while two-hour postprandial blood glucose (PBG) was measured 2 h after the beginning of fixed meals through finger blood tests. Diabetes was defined as FBG ≥ 7.0 mmol/L, PBG ≥ 11.1 mmol/L, glycated haemoglobin (HbA1c) ≥ 6.5% or the use of any glucose-lowering medication according to the American Diabetes Association [24]. According to the Guidelines on Prevention and Treatment of Dyslipidaemia for Chinese Adults [25], dyslipidaemia was defined as TG ≥ 2.3 mmol/L, TC ≥ 6.2 mmol/L, LDL-C ≥ 4.1 mmol/L, HDL-C < 1.0 mmol/L, or any lipid-lowering medication. The TyG index was denoted as ln[TG (mm/L)*fasting glucose (mm/L)/2]. The TG/HDL-C ratio was calculated as TG (mm/L) divided by HDL-C (mm/L).

### Assessment of baPWV

baPWV is a simple, noninvasive, automatic and widely used method in clinical practice and large population-based studies. The baPWV was measured with an Omron Colin BP-203RPE III device (Omron Health Care, Kyoto, Japan). After more than 5 min of rest in the supine position, 4 cuffs were wrapped around the bilateral brachia and ankles and then connected to a plethysmographic sensor and oscillometric pressure sensor. Semiconductor pressure sensors were used to assess the transmission time between the initial rises in both the brachial and tibial artery waves to record the pressure waveform. The distance between sampling points of baPWV was determined based on the height of the subjects. The time interval between the wave front of the brachial waveform and the ankle waveform was expressed as the time interval between the brachium and ankle (∆Tba). The final baPWV was calculated as

1 $${\text{baPWV }} = {\text{ }}\left( {{\text{La}} - {\text{Lb}}} \right)/\Delta {\text{Tba}}$$

La was the path length from the suprasternal notch to the ankle, and Lb was the path length from the suprasternal notch to the brachium using the following equations:

2 $${\text{La}} = 0.{\text{8129}} \times {\text{height of the participant }}\left( {{\text{in cm}}} \right) + {\text{12}}.{\text{328}}$$

3 $${\text{Lb = }}0.{\text{2195}} \times {\text{height of the participant }}\left( {{\text{in cm}}} \right) - {\text{2}}.0{\text{734}}$$

The detailed process has been described in a previous study [26]. The maximum value of baPWV on the left and right sides was chosen as the final result of baPWV. The baPWV was measured at baseline and at the follow-up visit. The baPWV change was calculated as the baPWV value at baseline minus the baPWV value at the last visit during follow-up. The baPWV change rate was calculated as the value of baPWV change divided by the time distance between baseline and last visit. The slope of baPWV was defined as the linear regression slope with examination year as the independent variable and multiple baPWV measurements as the dependent variable.

### Statistical analysis

Baseline characteristics are presented as the mean (standard deviation, SD), median [interquartile range, IQR] or number (percentage), as appropriate. Differences in baPWV change, baPWV change rate and slope of baPWV among groups were compared using the Kruskal–Wallis test.

Multivariate linear regression models were used to estimate the association of the TyG index and TG/HDL-C ratio with the absolute change, change rate and slope of baPWV. The TyG index and TG/HDL-C ratio were both analysed as continuous variables and categorized into quartiles. To adjust for potential confounding factors, three models were established as follows: model 1 adjusted for age and sex; model 2 adjusted for age, sex, BMI, smoking status, drinking status, physical activity, diabetes, dyslipidaemia, baPWV at baseline, and MAP at baseline and last follow-up; model 3 further adjusted for FBG (HDL-C if TG/HDL-C ratio analysed), triglyceride, PBG, LDL-C, eGFR, uric acid, homocysteine, and use of antidiabetic, lipid-lowering, or antihypertensive medications at baseline and follow-up. The regression coefficient and its 95% confidence interval (CI) are presented. Spearman’s correlation analyses of the TyG index and TG/HDL-C ratio with other common cardiometabolic risk factors were performed. To identify the interaction of insulin resistance indexes and other variables, the interactive terms were tested in the model.

All of the analyses presented above were conducted using R software (version 3.6.3). The difference was considered statistically significant at two-sided P < 0.05.

## Results

The final analysis included 1,895 individuals. During the follow-up period, a total of 986 recruited individuals attended health examinations twice, 612 had three examinations and 297 had four or five examinations. The mean age of the population was 61.90 ± 12.75 years, and 1,477 (77.9%) were men. At baseline, 1,013 (53.5%) participants were diagnosed with hypertension, and 882 (46.5%) were diagnosed with prehypertension, among which 335 participants progressed to hypertension at the last visit of follow-up. The median values of the baPWV change, the baPWV change rate and the slope of baPWV were 46 cm/s, 9 cm/s/year and 8.11 in the whole population, respectively, as shown in Table 1. The detailed characteristics according to the quartiles of the TyG index and TG/HDL-C ratio are presented in Additional file 1: Table S1 and Table S2.

**Table 1:** Characteristics of the study population

	Characteristics (N = 1895)
At baseline	
Age (years)	61.90 (12.75)
Sex (men, %)	1477 (77.9)
Occupation (n, %)	
Manager	392 (20.7)
Technician or professional	214 (11.3)
Worker	187 (9.9)
Retired	1102 (58.1)
BMI	26.52 (3.22)
Physical activity (n, %)	964 (50.9)
Smoking status: none/former/current (n, %)	1128 (59.6)/285 (15.0)/482 (25.4)
Current drinking (n, %)	844 (44.5)
SBP (mmHg)	139.64 (12.69)
DBP (mmHg)	78.67 (10.06)
MAP (mmHg)	98.99 (8.60)
Hypertension (n, %)	1013 (53.5)
Antihypertensive medication (n, %)	267 (26.4)
Diabetes (n, %)	323 (17.0)
Dyslipidaemia (n, %)	773 (40.8)
FBG (mmol/L)	5.79 (1.48)
PBG (mmol/L)	7.72 (2.61)
HbA1c (%)	5.94 (0.82)
Triglyceride (mmol/L)	1.86 (1.62)
Total cholesterol (mmol/L)	4.81 (0.98)
LDL-C (mmol/L)	3.11 (0.88)
HDL-C (mmol/L)	1.25 (0.32)
eGFR (mL/min per 1.73 m^2^)	90.88 (26.05)
Uric acid (μmol/L)	365.83 (84.67)
Homocysteine (μmol/L)	12.74 (7.66)
TyG index	1.42 [1.03,1.83]
TG/HDL-C ratio	1.22 [0.78,1.96]
baPWV (cm/s)	1538.00 [1392.00,1769.50]
At last visit of follow up	
baPWV (cm/s)	1603.00 [1412.00,1815.50]
Change of baPWV (cm/s)	46.00 [− 251.00,309.50]
Change rate of baPWV (cm/s/year)	9.00 [− 57.22,64.29]
Slope of baPWV	8.11 [− 54.72,66.96]
Hypertension (n, %)	1348 (71.1)
Antihypertensive medication (n, %)	562 (41.7)
Antidiabetic medication (n, %)	151 (8.0)
Lipid-lowering medication (n, %)	273 (14.4)

Data are the mean (SD), median [IQR] or number (%)

BMI: body mass index; SBP: systolic blood pressure; DBP: diastolic blood pressure; MAP: mean arterial pressure; FBG: fasting blood glucose; PBG: postprandial blood glucose; HbA1c: glycated haemoglobin; HDL-C: high-density lipoprotein cholesterol; LDL-C: low-density lipoprotein cholesterol; eGFR: estimated glomerular filtration rate; TyG: triglyceride glucose; baPWV: brachial-ankle pulse wave velocity

**Fig. 2:**
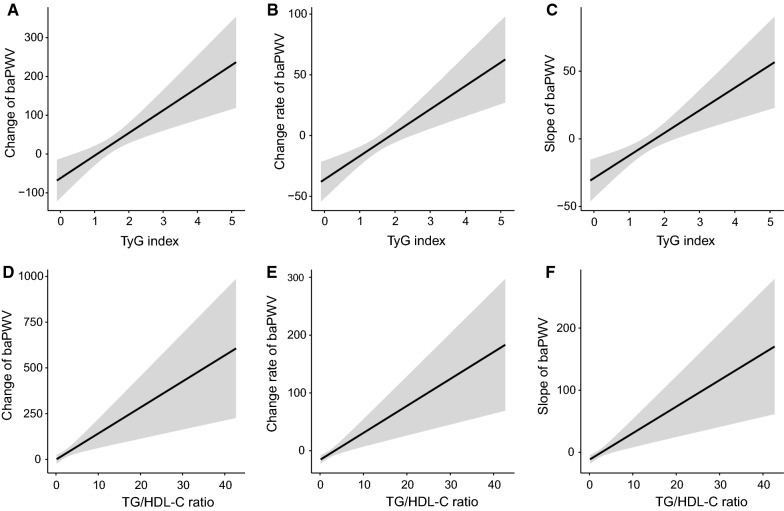
The regression line of the TyG index and TG/HDL-C ratio with artery stiffness progression. **A** TyG index and baPWV change; **B** TyG index and baPWV change rate; **C** TyG index and slope of baPWV; **D** TG/HDL-C ratio and baPWV change; **E** TG/HDL-C ratio and baPWV change rate; **F** TG/HDL-C ratio and slope of baPWV

**Fig. 3:**
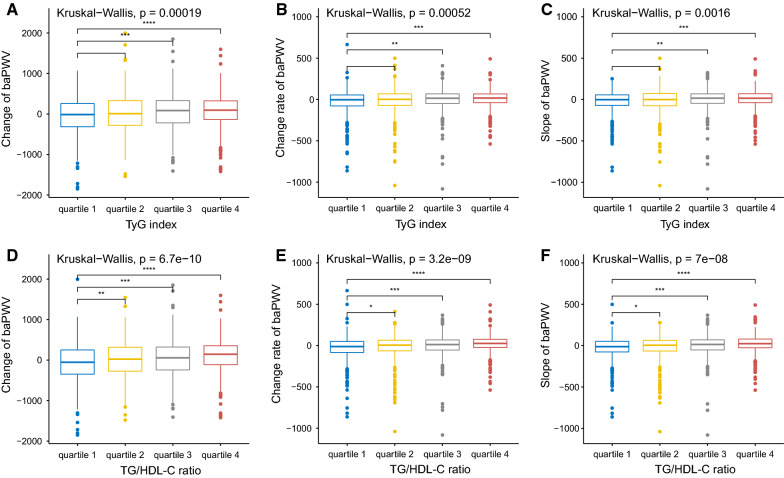
The distributions of the absolute change, change rate and slope of baPWV among the quartile groups according to the TyG index (**A**–**C**) and TG/HDL-C ratio (**D**–**F**)

**Fig. 4:**
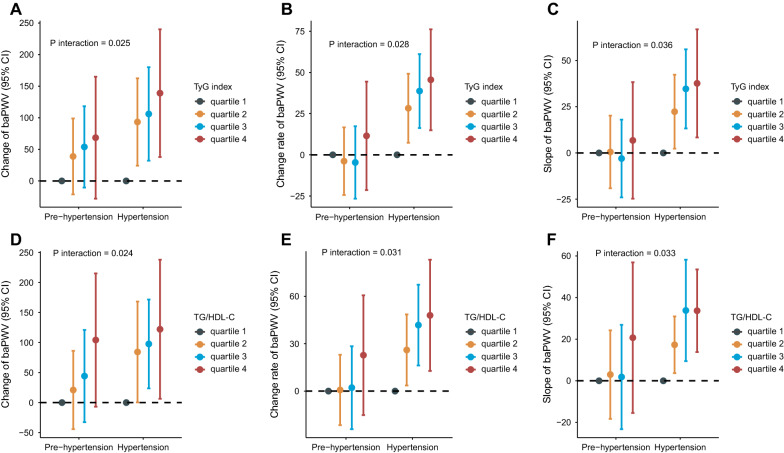
Associations of the TyG index and TG/HDL-C ratio with the absolute change, change rate and slope of baPWV in prehypertensive and hypertensive populations*. *Adjusted for age, sex, BMI, smoking status, drinking status, physical activity, diabetes, dyslipidaemia, baPWV at baseline, FBG (HDL-C if TG/HDL-C ratio analysed), triglyceride, PBG, LDL-C, eGFR, uric acid, homocysteine, MAP at baseline and follow up, and use of antidiabetic, lipid-lowering, or antihypertensive medications at baseline and follow up

We observed linear and positive associations of the TyG index and TG/HDL-C ratio with the baPWV change, baPWV change rate and slope of baPWV, as shown in Fig. 2. In the fully adjusted model (model 3), a one-unit increase in the TyG index was associated with a 149.6 cm/s increase in baPWV change, a 40.4 cm/s/year increase in the baPWV change rate, and a 34.9 cm/s increase in the baPWV slope. The average increases of baPWV change, change rate and slope, comparing participants in the highest quartile versus the lowest of the TyG index, were 129.5 cm/s (P for trend: < 0.001), 37.6 cm/s/year (P for trend: < 0.001), and 30.6 (P for trend: 0.002), respectively. Similarly, a one-unit increase in the TG/HDL-C ratio was associated with a 37.1 cm/s increase in baPWV change, a 9.8 cm/s/year increase in the baPWV change rate, and an 8.0 cm/s increase in the baPWV slope. The average increases in baPWV change, change rate and slope, comparing participants in the highest quartile versus the lowest quartile of the TG/HDL-C ratio, were 133.4 cm/s (P for trend: 0.002), 43.5 cm/s/year (P for trend: 0.001), and 33.5 (P for trend: 0.004), respectively. The detailed regression results are shown in Tables 2 and 3. The distributions of baPWV change, baPWV change rate and slope of baPWV among the quartile groups according to the TyG index are shown in Fig. 3A–C. The distributions of baPWV parameters among the quartile groups according to the TG/HDL-C ratio are shown in Fig. 3D–F. To identify the significant interaction of the TyG index and TG/HDL-C ratio with other covariates, such as age, sex, BMI, diabetes, dyslipidaemia, kidney function and life habits, we tested all of the interaction terms in the fully adjusted model (if not stratified). We found that only the associations of the TyG index and TG/HDL-C ratio with baPWV parameters were significantly different between prehypertensive and hypertensive populations. The strength of the associations of the TyG index and TG/HDL-C ratio with arterial stiffness progression were dominant in the hypertensive population, as shown in Additional file 1: Table S3 and Fig. 4. In addition, we fitted the joint TyG index and TG/HDL-C ratio in the fully adjusted model to evaluate the joint association with artery stiffness progression. There was a stronger association between the TyG index and the absolute change, change rate and slope of baPWV than the TG/HDL-C ratio, as shown in Additional file 1: Table S4.

**Table 2:** Association of the TyG index with the absolute change, change rate and slope of baPWV

	model 1			model 2			model 3		
	β	95% CI	P value	β	95% CI	P value	β	95% CI	P value
Change of baPWV (cm/s)									
TyG (continuous)	32.034	4.642–59.425	0.022	57.119	22.835–91.402	0.001	149.582	90.823–208.342	< 0.001
Quartile 2 (ref: quartile 1)	59.745	10.509–108.982	0.017	61.086	12.582–109.59	0.014	76.297	28.63–123.964	0.002
Quartile 3	85.613	36.416–134.81	0.001	93.631	43.889–143.373	< 0.001	102.562	52.08–153.044	< 0.001
Quartile 4	74.957	25.494–124.42	0.003	98.555	37.584–159.526	0.002	129.525	58.723–200.326	< 0.001
P for trend			0.002			< 0.001			< 0.001
Change rate of baPWV (cm/s/year)									
TyG (continuous)	11.892	3.415–20.368	0.006	17.974	7.307–28.642	0.001	40.372	21.853–58.891	< 0.001
Quartile 2 (ref: quartile 1)	11.870	-3.385–27.125	0.127	12.122	-2.986–27.229	0.116	16.529	1.507–31.551	0.031
Quartile 3	20.615	5.373–35.858	0.008	22.642	7.149–38.135	0.004	24.390	8.481–40.299	0.003
Quartile 4	24.616	9.291–39.941	0.002	30.750	11.76–49.741	0.002	37.607	15.294–59.919	0.001
P for trend			0.001			0.001			< 0.001
Slope of baPWV									
TyG (continuous)	9.570	1.538–17.601	0.020	14.238	4.121–24.355	0.006	34.874	17.238–52.51	< 0.001
Quartile 2 (ref: quartile 1)	10.986	-3.469–25.441	0.136	10.888	-3.439–25.215	0.137	14.872	0.57–29.175	0.042
Quartile 3	18.739	4.296–33.182	0.011	20.092	5.399–34.785	0.007	22.163	7.016–37.31	0.004
Quartile 4	20.126	5.604–34.647	0.007	23.648	5.639–41.658	0.010	30.576	9.332–51.82	0.005
P for trend			0.004			0.003			0.002

Model 1: Adjusted age and sex; Model 2: Age, sex, BMI, smoking status, drinking status, physical activity, diabetes, dyslipidaemia, baPWV at baseline, and MAP at baseline and follow-up; Model 3: Model 2 plus FBG, triglyceride, PBG, LDL-C, eGFR, uric acid, homocysteine, and use of antidiabetic, lipid-lowering, or antihypertensive medications at baseline and follow-up

**Table 3:** Association of TG/HDL-C ratio with the absolute change, change rate and slope of baPWV

	Model 1			Model 2			Model 3		
	β	95% CI	P value	β	95% CI	P value	β	95% CI	P value
Change of baPWV (cm/s)									
TG/HDL-C ratio (continuous)	6.795	− 1.193 to 14.784	0.096	4.934	− 3.684 to 13.552	0.062	37.057	7.85–66.264	0.013
Quartile 2 (ref: quartile 1)	70.320	21.037–119.604	0.005	68.883	19.964–117.802	0.006	75.317	24.101–126.534	0.004
Quartile 3	83.153	34.299–132.007	0.001	89.453	38.544–140.362	0.001	84.036	25.516–142.556	0.005
Quartile 4	131.912	82.542–181.282	< 0.001	142.938	78.525–207.35	< 0.001	133.420	51.949–214.891	0.001
P for trend			< 0.001			< 0.001			0.002
Change rate of baPWV (cm/s/year)									
TG/HDL-C ratio (continuous)	2.587	0.115–5.06	0.040	1.880	− 0.801 to 4.562	0.169	9.768	0.547–18.989	0.038
Quartile 2 (ref: quartile 1)	14.121	− 1.149 to 29.392	0.070	13.621	− 1.616 to 28.857	0.080	19.486	3.325–35.646	0.018
Quartile 3	23.979	8.842–39.117	0.002	25.543	9.687–41.399	0.002	29.615	11.15–48.08	0.002
Quartile 4	38.237	22.94–53.534	< 0.001	40.369	20.307–60.431	< 0.001	43.511	17.804–69.217	0.001
P for trend			< 0.001			< 0.001			0.001
Slope of baPWV									
TG/HDL-C ratio (continuous)	2.253	− 0.089 to 4.595	0.060	4.507	1.425–6.048	0.045	7.973	0.801–22.317	0.035
Quartile 2 (ref: quartile 1)	12.212	− 2.264 to 26.687	0.098	11.300	− 3.154 to 25.754	0.126	15.465	0.078–30.852	0.049
Quartile 3	21.636	7.287–35.986	0.003	21.977	6.935–37.019	0.004	24.296	6.715–41.877	0.007
Quartile 4	33.088	18.587–47.589	< 0.001	32.178	13.146–51.21	0.001	33.496	9.02–57.972	0.007
P for trend			< 0.001			< 0.001			0.004

Model 1: adjusted age and sex; Model 2: age, sex, BMI, smoking status, drinking status, physical activity, diabetes, dyslipidaemia, baPWV at baseline, and MAP at baseline and follow-up; Model 3: Model 2 plus HDL-C, triglyceride, PBG, LDL-C, eGFR, uric acid, homocysteine, and use of antidiabetic, lipid-lowering, or antihypertensive medications at baseline and follow-up

The correlation coefficients of the TyG index and TG/HDL-C ratio with other common cardiometabolic risk factors are described in Additional file 1: Figure S1. There were weak correlations of the TyG index and TG/HDL-C ratio with BMI, SBP, DBP, FBG, PBG, HbA1c, TC, LDL-C, serum uric acid, eGFR and homocysteine (Spearman’s coefficients less than 0.5), apart from TG and HDL-C.

## Discussion

In this prospective cohort study, we found that a higher TyG index and TG/HDL-C ratio were associated with a higher risk of arterial stiffness progression in the hypertensive population, during a median follow-up of 4.71 years. The observed associations were still significant after adjusting for important confounding factors, including age, BMI, blood pressure, medication use, lifestyle habits, serum uric acid, serum homocysteine, eGFR, diagnosis of diabetes and dyslipidaemia, which are traditional cardiometabolic risk factors and are related to arterial stiffness progression. TyG index and TG/HDL-C ratio deserve more attention in clinical practice for preventing artery stiffness and other cerebrocardiovascular complications in the hypertensive population.

Artery stiffness is a severe adverse event in the hypertensive population. On the other hand, arterial stiffness can lead to other macro- and microvascular complications and a series of organic damages, such as cerebrovascular diseases and renal damage [3, 27–29]. Moreover, research has found that some biological factors precede arterial stiffness progression in patients with hypertension, such as the duration of diabetes mellitus [30] and sodium sensitivity or resistance [31]. Therefore, identifying early and reliable related factors associated with artery stiffness in the hypertensive population is of great importance. The TyG index and TG/HDL-C ratio have been reported to be associated with artery stiffness in the general population in previous studies. However, evidence in prehypertensive and hypertensive populations is scarce, except for the study by Li et al. [20], which reported that the TyG index is associated with evaluated baPWV in a hypertensive population based on a cross-sectional design. Our study supplemented the evidence that the TyG index and TG/HDL-C ratio are associated with artery stiffness progression for the first time. We found that a higher TyG index and TG/HDL-C ratio were associated with an increased baPWV change, baPWV change rate and slope of baPWV in the hypertensive population based on a prospective cohort design.

The TyG index and TG/HDL-C ratio, have been of increasing interest at present. The TyG index and TG/HDL-C ratio have high sensitivity and specificity for the diagnosis of insulin resistance compared with the hyperinsulinaemic-euglycaemic clamp and homeostatic model assessment in many populations [32, 33]. These two indicators have a high possibility of being easily applied in clinical practice for the early detection of IR, arterial stiffness and other diseases. Previous studies have also demonstrated the association of the TyG index with cardiovascular, cerebrovascular and other metabolic diseases. In 2014, Fedchuk et al. [34] measured five steatosis biomarkers, including the TyG index, fatty liver index (FLI), NAFLD liver fat score (NAFLD-LFS), hepatic steatosis index (HSI) and visceral adiposity index (VAI), and found that all five steatosis biomarkers could contribute to the early diagnosis of steatosis and were correlated with IR. In the Northern Shanghai Study, Zhao et al. [17] concluded that an elevated TyG index was significantly related to arterial stiffness and nephric microvascular damage, which supported the clinical application of the TyG index for the assessment of vascular damage. In recent years, several studies have also reported that the TyG index may predict several cardiovascular diseases, including acute coronary syndromes, symptomatic CHD and ischaemic stroke [35–39]. For the TG/HDL-C ratio, in an 8-year Japan Diabetes Complications Study, Hirohito Sone et al. [40] evaluated conventional lipid variables, such as TG, non-HDLC, TC/HDL-C ratio, LDL-C/HDL-C ratio, and TG/HDL-C ratio, for a relationship with CHD. According to their analyses, all of these variables could predict CHD events in men and women. Marcello et al. [41] carried out a cross-sectional study to investigate the association between IR and TG/HDL-C with CHD and concluded that HOMA-IR and TG/HDL-C are positively associated with CHD and may be useful as high-specificity indicators of CHD for risk stratification. These studies indicate that the TyG index and TG/HDL-C ratio are promising markers for future screening of metabolic diseases.

In the current study, we found that the evaluated TyG index and TG/HDL-C ratio were independently associated with the progression of artery stiffness in the hypertensive population, while a significant association was not observed in the prehypertensive population. In a previous study [42], blood pressure and the hypertensive state itself were reported to worsen the progression of arterial stiffness. In a review analysis [43], age and high blood pressure were the two main determinants of arterial stiffness. The findings in our study imply that the interaction between hypertensive status and insulin resistance leads to arterial stiffness progression, which means that people with hypertension should pay close attention to insulin resistance indexes to prevent artery stiffness. Although not completely elucidated, there are potential mechanisms linking the TyG index and TG/HDL-C ratio with arterial stiffness. Insulin resistance is related with endothelial dysfunction, coagulation dysfunction, oxidative stress and inflammation, and cardiac concentric remodeling [44]. Hyperglycemia and hyperinsulinemia, usually accompanied by hyperlipidemia, are characterized by increased intracellular calcium concentration, increased collagen and advanced glycation end products, fibrosis and cellular hypertrophy, which reduce arterial elasticity through arterial remodeling especially in the hypertensive population, and lead to arterial stiffness [45, 46].

In addition, the TyG index and TG/HDL-C ratio, derived from fasting glucose, triglycerides and HDL-C, showed weak correlations with other components of metabolic syndrome, which implies that the TyG index and TG/HDL-C ratio might precede the incidence of the insulin resistance components [47]. Therefore, the TyG index and TG/HDL-C ratio may be early biomarkers of insulin resistance and other metabolic diseases, which warrants further validation in other studies.

The strengths of the present study include the prospective cohort design to explore the association of the TyG index and TG/HDL-C ratio with the progression of artery stiffness in prehypertensive and hypertensive populations, the adjustment of the potential confounding factors, and handling the TyG index and TG/HDL-C ratio as both continuous variables and categorical variables to enhance the reliability of our findings. However, the results should be interpreted in the context of some limitations. First, the sample size was relatively small, and the 95% CI of the estimated effect was wide. In the prehypertensive population, we only observed the evaluated tendency of baPWV change, baPWV change rate and slope of baPWV without statistical significance. The observed results in this single-centre study need further validation to generalize the associations in other populations and to evaluate the underlying biological mechanism between insulin resistance and artery stiffness progression. Second, the use of antihypertensive medication was considered in this analysis. However, we failed to collect the specific types of antihypertensive agents, given that different antihypertensive medications could have different influences on baPWV measurements. Data on antiplatelet medication were unavailable. Third, we analysed the association of the TyG index and TG/HDL-C ratio at baseline with artery stiffness progression in the current study, and evaluation of the dynamic TyG index and TG/HDL-C ratio was needed with multiple measurements and related methods, such as trajectory analysis.

## Conclusion

In summary, our findings indicate that the TyG index and TG/HDL-C ratio are significantly associated with arterial stiffness progression in hypertensive population, not in prehypertensive population. Monitoring the TyG index and TG/HDL-C ratio deserves more attention in clinical practice for the early prevention of arterial stiffness progression and other vascular complications of hypertension.

## Supplementary Information

**Additional file 1: Table S1.** Baseline characteristics by quartile groups of TyG index. **Table S2.** Baseline characteristics by quartiles of TG/HDL-C ratio. **Table S3.** The association of insulin resistance indexes and arterial stiffness progression in prehypertensive and hypertensive populations. **Table S4.** Joint relationship of TyG index and TG/HDL-C ratio with the absolute change, change rate and slope of baPWV. **Figure S1.** The correlation of TyG index and TG/HDL-C ratio with the cardio-metabolic risk factors.

## Data Availability

The datasets used and/or analysed during the current study are available from the corresponding author (Dr. Lixin Tao) on reasonable request.

## Abbreviations

TyG: Triglyceride glucose index
TG/HDL-C: Triglyceride to high-density lipoprotein cholesterol ratio
baPWV: Brachial-ankle pulse wave velocity
IR: Insulin resistance
BHMC: Beijing health management cohort
