# Supplementary material for: Association of TyG index and TG/HDL-C ratio with arterial stiffness progression in a non-normotensive population
Source: Cardiovasc Diabetol. 2021 Jul 6;20:134. doi: 10.1186/s12933-021-01330-6 (PMC8262008; doi:10.1186/s12933-021-01330-6)

Supplementary Table S1: Baseline characteristics by quartile groups of TyG index.

	quartile 1 (n=474)	quartile 2 (n=474)	quartile 3 (n=477)	quartile 4 (n=470)	P value
Age (year)	61.04(12.64)	60.86(12.82)	62.44(12.36)	63.28(13.05)	0.008
Sex (Men, %)	374(78.9)	384(81.0)	367(76.9)	352(74.9)	0.129
BMI	25.17(3.14)	26.22(2.95)	26.93(2.95)	27.69(3.15)	<0.001
Physical activity (n, %)	258(54.4)	232(48.9)	240(50.3)	234(49.8)	0.336
Smoking status (n, %)					
never smoking	274(57.8)	264(55.7)	306(64.2)	284(60.4)	0.056
current smoker	114(24.1)	139(29.3)	109(22.9)	120(25.5)	
former smoker	86(18.1)	71(15.0)	62(13.0)	66(14.0)	
Current drinking (n, %)	187(39.5)	214(45.1)	222(46.5)	221(47.0)	0.072
SBP (mmHg)	139.26(11.59)	139.46(13.51)	138.75(12.59)	140.71(12.90)	0.105
DBP (mmHg)	76.08(9.81)	76.80(9.13)	79.47(9.78)	82.49(10.07)	<0.001
MAP (mmHg)	97.14(8.02)	97.69(7.90)	99.23(8.36)	101.89(9.13)	<0.001
Hypertension (n, %)	261(55.1)	252(53.2)	236(49.5)	264(56.2)	0.176
Antihypertensive medication (n, %)	89(18.8)	78(16.5)	58(12.2)	42(8.9)	<0.001
Diabetes (n, %)	36(7.6)	51(10.8)	83(17.4)	153(32.6)	<0.001
Dyslipidemia (n, %)	59(12.4)	113(23.8)	178(37.3)	423(90.0)	<0.001
FBG (mmol/L)	5.22(0.63)	5.47(0.79)	5.77(1.19)	6.72(2.25)	<0.001
PBG (mmol/L)	7.01(2.01)	7.22(1.98)	7.67(2.48)	8.88(3.45)	<0.001
HbA1c (%)	5.73(0.47)	5.85(0.57)	5.91(0.73)	6.28(1.25)	<0.001
Triglyceride (mmol/L)	0.83(0.17)	1.27(0.20)	1.79(0.33)	3.56(2.47)	<0.001
Total cholesterol (mmol/L)	4.36(0.84)	4.74(0.93)	4.90(0.91)	5.23(1.04)	<0.001
LDL-C (mmol/L)	2.73(0.76)	3.17(0.86)	3.29(0.81)	3.25(0.95)	<0.001
HDL-C (mmol/L)	1.45(0.38)	1.29(0.27)	1.19(0.24)	1.07(0.24)	<0.001
eGFR (mL/min per 1.73 m^2)	92.17(28.15)	89.97(25.57)	91.85(25.36)	89.50(24.96)	0.294
Uric acid (μmol/L)	330.54(75.92)	361.84(78.19)	368.80(81.84)	402.43(86.87)	<0.001
Homocysteine (μmol/L)	12.59(7.32)	12.35(7.46)	13.07(8.40)	12.78(7.48)	0.518
TyG index	0.78[0.59,0.93]	1.22[1.13,1.32]	1.61[1.51,1.70]	2.20[1.98,2.47]	<0.001
TG/HDL-C ratio	0.58[0.45,0.76]	0.98[0.82,1.22]	1.55[1.23,1.84]	2.87[2.15,3.88]	<0.001
baPWV (cm/s)	1501.00[1385.25,1720.50]	1526.00[1388.00,1723.00]	1541.50[1400.25,1775.00]	1592.50[1406.50,1844.50]	0.001

Data are the mean(SD), median[IQR] or number (%).

BMI, body mass index; SBP, systolic blood pressure; DBP, diastolic blood pressure; MAP, mean arterial pressure; FBG, fasting blood glucose; PBG, postprandial blood glucose; HbA1c: glycated hemoglobin; HDL-C, high-density lipoprotein cholesterol; LDL-C, low-density lipoprotein cholesterol; eGFR, estimated glomerular fltration rate; TyG, triglyceride glucose; baPWV, brachial-ankle pulse wave velocity.

Supplementary Table S2: Baseline characteristics by quartiles of TG/HDL-C ratio.

	quartile 1 (n=475)	quartile 2 (n=466)	quartile 3 (n=483)	quartile 4 (n=471)	P value
Age (year)	60.48(12.48)	61.06(12.79)	62.32(12.70)	63.74(12.82)	<0.001
Sex (Men, %)	383(80.6)	352(75.5)	389(80.5)	353(74.9)	0.048
BMI	25.15(3.16)	26.30(2.95)	26.70(3.07)	27.85(2.96)	<0.001
Physical activity (n, %)	256(53.9)	243(52.1)	237(49.1)	228(48.4)	0.282
Smoking status (n, %)					
never smoking	274(57.7)	272(58.4)	297(61.5)	285(60.5)	0.128
current smoker	110(23.2)	129(27.7)	121(25.1)	122(25.9)	
former smoker	91(19.2)	65(13.9)	65(13.5)	64(13.6)	
Current drinking (n, %)	188(39.6)	212(45.5)	211(43.7)	233(49.5)	0.021
SBP (mmHg)	139.91(12.08)	139.11(13.03)	139.16(12.69)	139.99(12.91)	0.582
DBP (mmHg)	75.41(9.56)	77.21(9.57)	79.45(9.69)	82.73(9.77)	<0.001
MAP (mmHg)	96.91(8.06)	97.84(8.03)	99.35(8.11)	101.82(9.20)	<0.001
Hypertension (n, %)	265(55.8)	246(52.8)	248(51.3)	254(53.9)	0.566
Antihypertensive medication (n, %)	89(18.7)	74(15.9)	63(13.0)	41(8.7)	<0.001
Diabetes (n, %)	65(13.7)	67(14.4)	88(18.2)	103(21.9)	0.002
Dyslipidemia (n, %)	27(5.7)	99(21.2)	201(41.6)	446(94.7)	<0.001
FBG (mmol/L)	5.57(1.22)	5.62(1.33)	5.78(1.24)	6.19(1.94)	<0.001
PBG (mmol/L)	7.43(2.58)	7.46(2.51)	7.69(2.47)	8.19(2.94)	<0.001
HbA1c (%)	5.88(0.70)	5.92(0.84)	5.92(0.73)	6.05(1.03)	0.01
Triglyceride (mmol/L)	0.84(0.20)	1.27(0.24)	1.77(0.35)	3.56(2.45)	<0.001
Total cholesterol (mmol/L)	4.48(0.87)	4.78(0.98)	4.81(0.94)	5.16(1.01)	<0.001
LDL-C (mmol/L)	2.75(0.78)	3.19(0.89)	3.27(0.83)	3.23(0.91)	<0.001
HDL-C (mmol/L)	1.56(0.34)	1.30(0.21)	1.14(0.19)	1.00(0.19)	<0.001
eGFR (mL/min per 1.73 m^2)	94.77(29.02)	88.67(25.01)	91.14(24.45)	88.87(25.06)	0.001
Uric acid (μmol/L)	327.64(77.30)	359.39(77.72)	373.05(78.76)	403.30(86.88)	<0.001
Homocysteine (μmol/L)	12.86(8.50)	12.10(6.66)	12.98(7.64)	12.84(7.76)	0.281
TyG index	0.80[0.59,1.00]	1.22[1.06,1.40]	1.58[1.42,1.73]	2.13[1.89,2.46]	<0.001
TG/HDL-C ratio	0.56[0.45,0.68]	0.97[0.88,1.08]	1.54[1.37,1.72]	2.88[2.32,3.88]	<0.001
baPWV (cm/s)	1477.00[1370.50,1655.50]	1536.00[1390.00,1746.00]	1537.50[1396.50,1758.00]	1634.00[1418.00,1874.50]	<0.001

Data are the mean(SD), median[IQR] or number (%).

BMI, body mass index; SBP, systolic blood pressure; DBP, diastolic blood pressure; MAP, mean arterial pressure; FBG, fasting blood glucose; PBG, postprandial blood glucose; HbA1c: glycated hemoglobin; HDL-C, high-density lipoprotein cholesterol; LDL-C, low-density lipoprotein cholesterol; eGFR, estimated glomerular fltration rate; TyG, triglyceride glucose; baPWV, brachial-ankle pulse wave velocity.

Supplementary Table S3: The association of insulin resistance indexes and arterial stiffness progression in prehypertensive and hypertensive populations.

	population	Change (cm/s)		Change rate (cm/s/year)		Slope	
		Coefficient*	P value	Coefficient*	P value	Coefficient*	P value
**TyG index**							
quartile 1	pre-hypertension						
quartile 2		38.798	0.206	-3.866	0.712	0.516	0.959
quartile 3		53.848	0.102	-4.630	0.680	-3.024	0.778
quartile 4		68.463	0.165	11.519	0.493	6.777	0.674
quartile 1	hypertension						
quartile 2		93.330	0.008	28.292	0.008	22.311	0.029
quartile 3		106.026	0.005	38.729	0.001	34.650	0.002
quartile 4		138.954	0.007	45.564	0.004	37.632	0.012
**TG/HDL-C ratio**							
quartile 1	pre-hypertension						
quartile 2		20.995	0.528	0.736	0.948	2.990	0.783
quartile 3		44.158	0.260	2.178	0.871	1.842	0.886
quartile 4		104.200	0.066	22.751	0.240	20.709	0.262
quartile 1	hypertension						
quartile 2		84.301	0.049	26.048	0.023	17.299	0.014
quartile 3		97.654	0.010	41.799	0.001	33.803	0.007
quartile 4		122.090	0.039	47.953	0.008	33.675	0.005

*adjusted for age, sex, BMI, smoking status, drinking status, physical activity, diabetes, dyslipidemia, baPWV at baseline, FBG (HDL-C if TG/HDL-C ratio analyzed), triglyceride, PBG, LDL-C, eGFR, uric acid, homocysteine, MAP at baseline and follow up, use of antidiabetic, lipid-lowering, antihypertensive medications at baseline and follow up.

Supplementary Table S4: Joint relationship of TyG index and TG/HDL-C ratio with the absolute change, change rate and slope of baPWV.

	Coefficient*	Se	95% CI	P value
**Change of baPWV (cm/s)**				
TyG index	127.657	36.929	55.276-200.039	0.001
TG/HDL-C ratio	-4.846	17.958	-40.042-30.351	0.787
**Change rate of baPWV (cm/s/year)**				
TyG index	39.392	11.658	16.541-62.243	0.001
TG/HDL-C ratio	0.104	5.669	-11.007-11.216	0.985
**Slope of baPWV**				
TyG index	33.607	11.101	11.849-55.366	0.003
TG/HDL-C ratio	0.413	5.398	-10.167-10.993	0.939

*adjusted for age, sex, BMI, smoking status, drinking status, physical activity, diabetes, dyslipidemia, baPWV at baseline, FBG (HDL-C if TG/HDL-C ratio analyzed), triglyceride, PBG, LDL-C, eGFR, uric acid, homocysteine, MAP at baseline and follow up, use of antidiabetic, lipid-lowering, antihypertensive medications at baseline and follow up.

Supplementary Figure S1: The correlation of TyG index and TG/HDL-C ratio with the cardio-metabolic risk factors.

	Spearman's coefficients		P value	
	TyG	TG/HDLC	TyG	TG/HDLC
BMI (kg/m²)	0.307	0.314	<0.001	<0.001
SBP (mmHg)	0.015	-0.027	1.000	1.000
DBP (mmHg)	0.257	0.274	<0.001	<0.001
MAP (mmHg)	0.207	0.198	<0.001	<0.001
FBG (mmol/L)	0.424	0.197	<0.001	<0.001
PBG (mmol/L)	0.240	0.118	<0.001	<0.001
HblAc	0.157	0.033	<0.001	1.000
TG (mmol/L)	0.855	0.849	<0.001	<0.001
TC (mmol/L)	0.326	0.229	<0.001	<0.001
LDLC (mmol/L)	0.223	0.186	<0.001	<0.001
HDLC (mmol/L)	-0.475	-0.718	<0.001	<0.001
Uric acid (umol/L)	0.301	0.340	<0.001	<0.001
eGFR (mL/min per 1.73 m²)	-0.007	-0.056	1.000	0.757
Homocysteine (μmol/L)	0.023	0.033	1.000	1.000

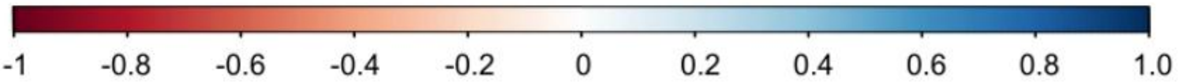

Supplement: Supplementary file 1 — Additional file 1: Table S1. Baseline characteristics by quartile groups of TyG index. Table S2. Baseline characteristics by quartiles of TG/HDL-C ratio. Table S3. The association of insulin resistance indexes and arterial stiffness progression in prehypertensive and hypertensive populations. Table S4. Joint relationship of TyG index and TG/HDL-C ratio with the absolute change, change rate and slope of baPWV. Figure S1. The correlation of TyG index and TG/HDL-C ratio with the cardio-metabolic risk factors. [file 12933_2021_1330_MOESM1_ESM.pdf]
